# Deep convolution stack for waveform in underwater acoustic target recognition

**DOI:** 10.1038/s41598-021-88799-z

**Published:** 2021-05-05

**Authors:** Shengzhao Tian, Duanbing Chen, Hang Wang, Jingfa Liu

**Affiliations:** 1 Big Data Research Center, University of Electronic Science and Technology of China, Chengdu, 611731 China; 2 The Research Base of Digital Culture and Media, Sichuan Provincial Key Research Base of Social Science, Chengdu, 611731 China; 3 Union Big Data Tech. Inc., Chengdu, 610041 China; 4 Guangzhou Key Laboratory of Multilingual Intelligent Processing, Guangdong University of Foreign Studies, Guangzhou, 510006 China; 5 School of Information Science and Technology, Guangdong University of Foreign Studies, Guangzhou, 510006 China

**Keywords:** Computer science, Information technology

## Abstract

In underwater acoustic target recognition, deep learning methods have been proved to be effective on recognizing original signal waveform. Previous methods often utilize large convolutional kernels to extract features at the beginning of neural networks. It leads to a lack of depth and structural imbalance of networks. The power of nonlinear transformation brought by deep network has not been fully utilized. Deep convolution stack is a kind of network frame with flexible and balanced structure and it has not been explored well in underwater acoustic target recognition, even though such frame has been proven to be effective in other deep learning fields. In this paper, a multiscale residual unit (MSRU) is proposed to construct deep convolution stack network. Based on MSRU, a multiscale residual deep neural network (MSRDN) is presented to classify underwater acoustic target. Dataset acquired in a real-world scenario is used to verify the proposed unit and model. By adding MSRU into Generative Adversarial Networks, the validity of MSRU is proved. Finally, MSRDN achieves the best recognition accuracy of 83.15%, improved by 6.99% from the structure related networks which take the original signal waveform as input and 4.48% from the networks which take the time-frequency representation as input.

## Introduction

In underwater acoustic target recognition, target radiated noise collected by hydrophones is used to identify targets. Due to the complexity of the ocean soundscape, collected radiated noise is always accompanied by a great deal of interferential noise. Because the collected noise is neither explicit nor semantic, it is difficult to label and classify. How to improve the performance of automatic detection and classification of acoustic radiated noise signal is still a challenge problem.

Generally, conventional underwater acoustic target recognition methods extract manual designed features to train the classifiers. Due to the huge effect on recognition performance, traditional methods pay more attention to the design and extraction of features. The conventional manual designed features mainly include waveform features^1–3^, wavelet features^4,5^, spectrum features^6–10^, and auditory features^11,12^. The characteristics of the underwater targets are described well by manual designed features from different perspectives. However, designing these features requires a great deal of prior knowledge of targets. For unknown targets and complex underwater soundscape, it is difficult to acquire enough prior knowledge. Hence, facing the unknown complex ocean acoustic field, manual designed features are not robust. Although preprocessing methods such as feature selection and feature fusion^13–17^ were alleviated the weak generalization ability of manual designed features to some extent, the inherent generalization ability problem of these features still cannot be solved radically. Besides, in traditional methods, designing classifiers and extracting features are relatively independent, so the designed features may not fit the classification models^18^. Briefly, the traditional methods have difficulty in adapting to the complex and changeable ocean environment. Thus, the underwater acoustic target recognition still mainly relies on well-trained sonar man^19^.

With the great progress of deep learning, deep neural networks have been successfully applied not only in pure visual and semantic perception but also in intelligent transportation^20,21^, intelligent industrie^22^ and intelligent security^23^. It becomes feasible that build and train an end-to-end deep neural network to identify underwater acoustic targets by extracting deep features automatically. Instead of using hand-engineered features as before, Cao et al.^24^ used Sparse Auto-Encoders to learn the hidden structure from the time-frequency (T-F) representation of underwater target acoustic signals in an unsupervised manner. The recognition performance was improved greatly compared with the traditional methods. Mello et al.^25^ used Stacked Auto-Encoders on the T-F representation for classification as well as detection of novelty categories that do not appear in the training set. Kamal et al.^26^ used a Deep Belief Network (DBN) to classify the underwater acoustic targets and the results confirmed the robustness of the approach in complex ambiences and the applicability of deep feature learning approaches for underwater target recognition. Yue et al.^27^ compared DBN to Convolutional Neural Network (CNN) using spectrogram as the input of networks, and the results showed that deep learning methods can achieve higher recognition accuracy. Yang et al.^28^ used a competitive learning mechanism to increase cluster performance during training of the deep network, and achieved higher accuracy than traditional methods. Wu et al.^29^ proposed a modified CNN based on typical CNN to classify the LOFAR (Low-Frequency Analysis and Recording) spectrogram. Cao et al.^30^ proposed a novel classification framework which combines the CNN architecture with the second-order pooling (SOP) to capture the temporal correlations from the T-F representation of the radiated acoustic signal and the proposed method yields an 8% improvement in classification accuracy over the state-of-the-art deep learning methods. Besides, salp swarm algorithm^31^ and chimp optimization algorithm were developed by Khishe et al.^32^ for training neural networks, and results showed that the newly proposed algorithm in most cases provides better or comparable performance. In application of Recurrent Neural Network (RNN), Yang et al.^33^ combined deep long short-term memory network (LSTM) and deep auto-encoder neural network (DAE). The LSTM model in the DAE was pre-trained via unsupervised learning. The proposed method achieved a better classification performance compared with only using DAE and LSTM. Yuan et al.^34^ proposed a multimodal deep learning method for the recognition of ship-radiated noise. Ship-radiated noise (acoustics modality) and visual observation of the ships (visual modality) are two different modalities that the multimodal deep learning method models on. Liu et al.^35^ proposed a one-dimensional convolutional neural network (1D-CNN) to recognize the line spectrums of Detection of Envelope Modulation on Noise (DEMON) spectrums of underwater target-radiated noise.

All deep learning models mentioned above took the time-frequency representation as the input of the networks, such as spectrogram, LOFAR spectrogram, Mel Frequency Cepstral Coefficient, DEMON spectrum and so on. By spectrogram calculation, the original signal was converted to the time-frequency domain with more explicit characteristics. Meanwhile, the information from waveform fine structure was lost. Besides, time-frequency representations are usually limited by the generation parameters, such as the window size of Fourier Transform (FT) and the hop length of FT window. On the one hand, it requires prior knowledge to determine the appropriate transform parameters, and the time and frequency resolution cannot reach the optimum simultaneously. On the other hand, once the parameters are determined, the resolution of the generated spectrogram is fixed accordingly. It causes the loss of other resolution information for the end-to-end model with fixed input size. It becomes the bottleneck of spectrogram-based methods. Naturally, modeling on original signal waveform directly as well as predicting targets in a whole model becomes the direction to break through the bottleneck of performance.

Hu et al.^36^ conducted feature extraction to original waveform of underwater sound signal by CNN and took the extracted features as the input features of extreme learning machine classifier. Recognition rate was greatly improved compared to the traditional methods. Shen et al.^37,38^ proposed auditory inspired convolutional neural networks trained from raw underwater acoustic signal. A bank of trainable gammatone filters simulated the cochlea filter banks to extract features from original signal. The classification performance had been improved. Yang et al.^19^ designed a bank of multiscale deep convolution filters to decompose raw time domain signal into signals with different frequency components and made an improvement by refining the fusion and classification layers of depth characteristics. It achieved a classification accuracy of 81.96%. Recently, Shen et al.^18^ continued to improve their model by using Inception-Resnet^39^ as deep architecture for classification and adding an auxiliary classifier to recalibrate auditory features extracted from trainable gammatone filter banks. The model achieved 87.2% recognition accuracy on four ship types and ocean background noise. Hu et al.^40^ designed auditory perception inspired time-dilated convolution neural network (ATCNN) based on depthwise separable convolution and time-dilated convolution. Intra-class and inter-class information can be fully used for classification. For generative tasks, the Generative Adversarial Networks (GAN) have been proved to be effective in synthesis and restoration of voices^41^ and pictures^42^. GAN improves the ability of sample generation through adversarial learning between a generator and a discriminator. A good generator can be used to extend the training dataset, and improve the generalization and robustness of recognition model. In underwater acoustic target recognition, Jin et al.^43^ utilized GAN to extend the dataset by generating LOFAR spectrogram, and improved the performance of classification. However, due to the complexity of the signal waveform and the instability of the training process, few works have been done on using GAN to synthesize the underwater acoustic signal waveform so far.

Building and training an end-to-end neural network to classify underwater acoustic targets have been gradually adopted by researchers. However, in previous work, most of models have few layers and utilize large kernels at the beginning of the network for learning and extracting features. The network structures are imbalanced, because the convolutional layers with large kernels consume most of the memory. The networks are more dependent on the front architecture. It also leads to the separation of feature extraction and classification imperceptibly, and makes researchers gradually pay more attention on designing front of networks just like conventional methods. Deep convolution stack is a kind of network frame with flexible and balanced structure. It has good robustness since it does not depend on a particular part of the network. Automatic Machine Learning (AutoML) techniques such as Neural Architecture Search (NAS) can be easily applied on deep convolution stack networks due to the regularity of structure. In fact, deep convolution stack networks have not been explored well in underwater acoustic target recognition, even though such deep convolution stack networks like ResNet^44,45^, Inception^39^ and DenseNet^46^ have been proved to be flexible and effective in visual understanding and natural language processing. The reason for not using the deep convolution stack networks may be the lack of a basic stack unit which is effective on perceiving the one-dimensional signal waveform of underwater sound. Unlike the speech audio, underwater sound is more irregular and random due to the diversity and uncertainty of sound source. Therefore, the general deep network structure may not be suitable for underwater acoustic target recognition.

In this paper, we focus on exploring appropriate network structure of deep convolution stack for perception underwater target radiated noise, giving full play to the automatic feature learning and extraction capabilities of deep neural networks. To model the one-dimensional original waveform, a multiscale residual unit (MSRU) is proposed inspired by DRSN^47^ and deep neural network (DNN)^48,49^. Soft-thresholding proposed from DRSN^47^ and large convolution kernel used in^48,49^ are combined as the initial design inspiration. Multiscale convolution is used to replace the core convolution in DRSN. By stacking MSRU, we present a multiscale residual deep neural network (MSRDN) for underwater acoustic target recognition, which takes original waveform as input. Dataset acquired in a real-world scenario is used to verify the effectiveness of our model. MSRDN achieves the best recognition accuracy of 83.15%, improved by 6.99% from the structure related networks which take the original signal waveform as input and 4.48% from the networks which take the time-frequency representation as input. MSRDN can improve the performance in underwater acoustic target recognition. Besides, to verify the MSRU in a different perspective, we add MSRU into Generative Adversarial Networks by replacing the core convolutional layers in BigGAN^42^ and WaveGAN^41^. The results of experiments have proved the effectiveness of the MSRU.

The main contributions of this paper are summarized as follows:

- For original signal waveform of the underwater acoustic target, a multiscale residual unit (MSRU) is proposed. The receptive field of the model has been improved. The results of classification and generative experiments prove the effectiveness of the MSRU.
- By stacking MSRU, a multiscale residual deep neural network (MSRDN) for underwater acoustic target recognition is presented. Comparative experiments results show that MSRDN improves the classification performance of underwater acoustic targets.
- For generative tasks, we preliminarily explore using Generative Adversarial Networks to synthesize underwater acoustic signal waveform. Two advanced GAN models are modified by using MSRU and the performance of them are improved.
- An approach of making underwater acoustic dataset is summarized in this paper. Detailed descriptions and analyses are correspondingly carried out. Several affecting factors that may impact the recognition performance are proposed.

The remains of the paper is organized as follows. The proposed MSRU and MSRDN including two initial design inspirations are detailedly described in Methods. The manufacturing process of the dataset from a real-world scenario is introduced in Data Description Section. The experimental results and analyses are given in Results Section. Conclusions and future works are drawn in Discussions Section.

## Methods

### Multiscale residual unit

Inspired by DRSN^47^ and deep neural network (DNN)^48,49^, multiscale residual unit (MSRU) is proposed in this paper. Concretely, we are attracted to the soft-thresholding proposed in DRSN^47^ and large convolution kernel used in^48,49^.

#### Soft-thresholding

Soft-thresholding has been used in many signal denoising methods. The function of soft thresholding can be expressed as

1 $$\begin{aligned} f(x)= {\left\{ \begin{array}{ll} x-\tau , &{} x > \tau \\ 0, &{} -\tau \le x\le \tau \\ x+\tau , &{} x < -\tau \\ \end{array}\right. } , \end{aligned}$$

where *x* is the input feature, *f*(*x*) is the output feature, and $\tau$ is the threshold. Instead of setting the negative features to zero in ReLU activation function, soft thresholding sets the near-threshold features to zeros, so that useful negative features can be preserved. Different from image recognition and natural language processing, the negative features or the negative correlations of the one-dimensional signal are also important in classification due to the time variability of the signal. It is helpful for the model to improve the perception ability of one-dimensional signal by retaining the negative feature and restraining the near zero feature.

**Figure 1:**
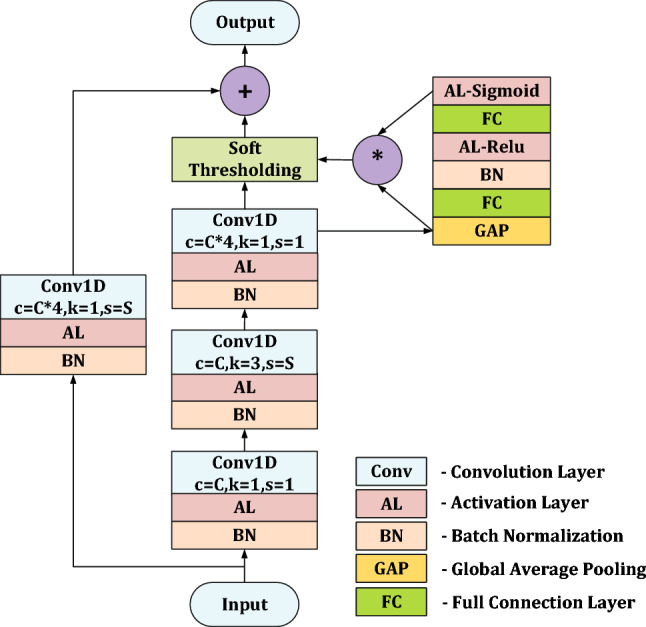
The basic unit of DRSN^47^. In convolution layer, parameter *c* is the convolutional out channel number, *k* is the kernel size of convolution, and *s* is the stride of convolution.

Compared with the basic unit used in ResNet^45^, soft-thresholding is inserted as nonlinear transformation layers into the deep architectures to eliminate unimportant features in DRSN^47^. Without manual presetting, the threshold can be learn automatically by a nonlinear transformation mechanism. Figure 1 shows the basic unit of DRSN^47^. Firstly, we define a group of computational operations including a batch normalization, an activation and a convolution. The information in unit flows through two paths. one is the main information path and another is the shortcut information path. There are three groups of computational operations sequentially in the main information path. Following computational groups is a Soft-thresholding layer which learned the threshold by the nonlinear transformation structure in top right corner. To match the output shapes of two paths, there is one computational group in the shortcut information path. Hyper-parameter *C* is set as the number of processing channels for the entire unit. Hyper-parameter *S* is set as the stride of both the middle convolution in main path and the convolution in shortcut path. The channels of input data will be transformed to *C* by the first computational group in main information path and then the channels will expand to 4*C* by the last computational group in main information path. After Soft-thresholding layer, the output of the main information path and the shortcut information path will be added to form the final output.

#### Large convolution kernel

As we know, 3 × 3 convolutional kernel has been proved to be the most efficient kernel for image recognition. The 3 × 3 convolutional kernel could capture fine features of image very well. However, for one-dimensional signal waveform, the appropriate kernel size may be different due to the superposition of different periodic signals and the difference of sampling frequency. Hence, the kernel size of 3 may be not the good choice for convolution on one-dimensional signal waveform. In fact, for signal with sampling frequency being 8 kHz or 16kHz, only using kernel size of 3 will result in insufficient receptive field of model and require a very deep convolution layer stack to perceive the features of waveform. The DNN in^48,49^ took the kernel size of 16 as the filter length. Shen et al.^18^ took the kernel size of 100 as the gammatone filter length. Hu et al.^36,40^ took the kernel size of 204, 12 and 14. Li et al.^50^ took the kernel size of 128 as the filter length. All these works demonstrate that for one-dimensional signal waveform, better performance can be achieved if we increase convolutional kernel size appropriately.

However, due to the limitation of storage and computing resource, it is difficult to apply deep convolution stack with large convolutional kernel size. With limited resources, it is feasible to use large kernel size to construct convolution stack with few layers or to use small kernel size to construct convolution stack with deep layers. In underwater acoustic target recognition, the first strategy was taken by Shen et al.^18,37,38^, Yang et al.^19^ and Hu et al.^36,40^ They all used large convolutional kernel at the front of the network to form a filter bank. Then, typical convolution stack with small kernel size was used to classify the output of the filter bank. The second strategy was taken by^47–49^ in highly noised vibration signals and computerized electrocardiogram identification. They used medium size convolutional kernel through the network from start to end to construct deep convolution stack network. The second strategy has also been proved to be effective on one-dimensional signal waveform. In fact, deep convolution stack has not been explored well in underwater acoustic target recognition. Hence, in this paper, we will take the second strategy on underwater acoustic target recognition. At the same time, in order to avoid the inadequacy problem of small convolutional kernels, we consider using multiscale convolutional kernels to enhance the receptive field of the model.

#### Implementation of multiscale residual unit

**Figure 2:**
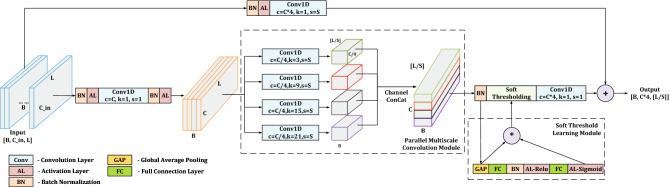
The structure of Multiscale Residual Unit (MSRU). Two hyper-parameters *C* and *S* will determine the output shape. The shape of input data is $[B,C\_in,L]$ in which *B* represents batch size, $C\_in$ represents channel number, and *L* represents data length. The shape of output data is $[B,C\times4,L/S]$. Hyper-parameters *S* is usually set to 1 or 2. Parallel multiscale convolution module consists of four convolutional layers with different kernel size and a channel concat operation. Soft Threshold Learning Module consists of a global average pooling layer and two fully connected nonlinear transformation layer. In every convolution layer, parameter *c* is the convolutional out channel number, *k* is the kernel size of convolution, and *s* is the stride of convolution. All the padding approach are “same”.

Inspired by DRSN^47^ and DNN^48,49^, Multiscale Residual Unit is presented in Fig. 2. MSRU is designed on the basis of deeper bottleneck architectures in ResNet^44^. Soft-thresholding and multiscale convolutional kernels are used to increase the perception ability for one-dimensional underwater acoustic signals. Using multiscale convolutional layers will be able to enhance the receptive field of the model. On a micro level, like the spectrum calculation, once the kernel size of convolution is determined, the resolution of the generated feature map is fixed accordingly. In contrast, multiscale convolutional layers will be able to generate feature maps with multiple resolutions and combine them. The inadequacy problem of small convolutional kernels can be avoided. On a macro level, because the convolutional layer usually processes on the output of the previous convolutional layer, the low resolution information will be delivered to the deep layer. Generating feature maps with multiple resolutions during forward propagation allows the model to capture more feature information, by combining and calculating layer by layer. The imbalance structure problem of large convolutional kernels can be solved.

In order to make the features balanced at each scale and maintain the multiscale sensitivity of the model, the number of channels processed at each layers in parallel multiscale convolution module should be equal. For the convenience of the channel calculations, four parallel layers of convolution will be used in MSRU. Besides, we conducted experiments on 2 and 8 layers of multiscale convolution module. The results did not exceed the model with 4 layers in module. Under the trade-off between resource and performance, we adopt the convolutional kernel with size of 3, 9, 15 and 21 in four parallel convolutional layers respectively.

Two hyper-parameters which will determine the output shape need to be set in advance. Hyper-parameter *C* is set as the number of processing channels for the entire unit. Hyper-parameter *S* is set as the stride of multiscale convolution as well as the stride of convolution in shortcut path. The information flows through two paths. one is the main information path and another is the shortcut information path. In main information path, the channels of input data will be transformed to *C* by the first convolutional layer. Then, four convolutional layers with different kernel size will extract features from different scales. Channel concat will be used to fusion the output features of each convolutional layer. Soft Threshold Learning Module will generate a threshold for every channels by nonlinear transformation. After filtered by the learned thresholds, the channels of feature will expand to 4*C* by the last convolutional layer. In shortcut information path, following by batch normalization and activation layer, one convolutional layer will be used to match the output shapes of main and shortcut paths. Finally, the output of the main and shortcut path will be added to form the final output.

Compared with the basic unit of DRSN^47^ (see Fig. 1), one dimensional convolution at the middle computational group in main path is replaced by four parallel convolutional layers with different kernel size. The number of output channels per convolutional layer is one quarter of the input channels. After channel concat, the size of output feature equals the size of basic unit in DRSN^47^. Therefore, only the multiscale convolution part needs extra parameters and computations. Besides, the activation layer before the last convolution layer will be replaced by soft-threshold layer. In this way, multiscale features could be filtered directly and the amount of computation and parameters required for soft-threshold layer could be reduced, because the whole process is performed before the channel promotion. All designs will significantly improve model receptive field with small increase in parameters.

Different from the Auditory perception inspired Deep Convolutional Neural Network (ADCNN)^19^, in which filters with different convolutional scales were only set up in the beginning of the network, MSRU with four different convolutional scales runs through the network from start to end. By varying the number of MSRU, the network can be adjusted more easily facing different environments. In addition, both the combination strategies of different convolutional kernel size and the number of MSRU can be used as entry points of network structure search.

### Multiscale residual deep neural network

**Figure 3:**
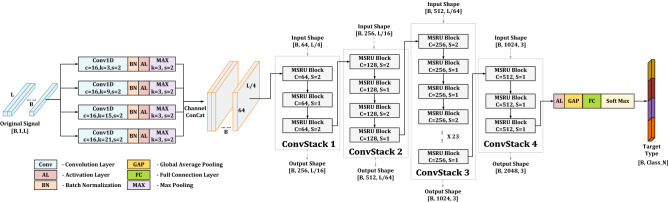
The structure of multiscale residual deep neural network (MSRDN). The shape of input data is [*B*, 1, *L*], in which *B* represents batch size and *L* represents data length. The shape of output is $[B,Class\_N]$, in which *B* represents batch size and $Class\_N$ represents the number of predicted categories. In the head of the network are four parallel convolution with different kernel size. the main body of MSRDN is stacked by MSRU. According to the difference of hyper-parameter *C*, all MSRUs will be divided into four convolution stacks. The convolution stacks can be directly connected to each other due to the independence and flexibility of MSRU.

Following the advanced structures of deep convolution stack networks, multiscale residual deep neural network (MSRDN) stacked by MSRU is constructed in Fig. 3. In the head of the network, four parallel convolutional layers with different kernel size will be performed firstly. The selection of kernel size is same as multiscale convolution module in MSRU. The reason for using the four parallel layers is to avoid the limitation by a fixed convolutional kernel size initially, and the multiscale features in low resolutions are ensured to the maximum extent. The output channel of each convolution layer is set to 16 and the stride of each convolution layer is set to 2. After bath normalization and activation layer, a max Pooling layer (kernel size = 3, stride = 2) will be used to reduce the output dimensions of each convolution. Then, channel concat will be used to fusion the output features. The shape of the feature map after fusion is [*B*, 64, *L*/4].

Based on structure of ResNet-101^44^, the main body of MSRDN is stacked by MSRU. According to the difference of hyper-parameter *C*, all MSRUs will be divided into four convolution stacks. The number of MSRUs in four stacks is distributed as [3, 4, 23, 3], and the hyper-parameter *C* is set as [64, 128, 256, 512] respectively. In each convolution stacks, the MSRUs are connected sequentially. The hyper-parameter *S* in each MSRU can be set to 2 or 1. If *S* is set to be 2, MSRU will perform once sub-sampling to the input. Otherwise, no sub-sampling will be performed. In our model, for two consecutive MSRUs in convolution stack 1 and convolution stack 2, *S* is set to 2 in prior MSRU and 1 in other unit. While for three consecutive MSRUs in convolution stack 3, *S* is set to 2 in prior unit and 1 in other two units. In convolution stack 4, *S* is set to 1 for all MSRUs.

At the end of the network, after a activation layer, a Global Average Pooling layer and a full connection layer will be used to generate result of prediction. Finally, the result of prediction will be transformed to a probability distribution by a soft-max layer. The shape of output is $[B,Class\_N]$, in which *B* represents batch size and $Class\_N$ represents the number of predicted categories.

Compared with the structure of ResNet-101^44^, MSRDN changes the initial convolution layer in the head of network into four parallel multiscale convolution layers. Then, general residual units in ResNet-101 are replaced by proposed MSRU. The unit distribution of each convolutional stack remains the same. With such modification of structure, MSRDN would automatically learn and extract multiscale features from underwater acoustic radiated noise. Meanwhile, the benefits of ResNet^44^ are preserved. Besides, MSRDN are not dependent on a particular part of structures, becaue MSRUs run through the main body of MSRDN from start to end. The network structure becomes more regular and balanced.

## Data description

### Data source

The sample data of underwater targets used in this paper are collected from Ocean Networks Canada (https://oceannetworks.ca). Specifically, we choose the signals recorded by Ocean Sonics icListen AF Hydrophone 2523 deployed at Latitude $49.080811^{\circ }$, Longitude $-123.3390596^{\circ }$ and 144 meters below sea level. The dates of data acquisition are from 24 Jun, 2017 to 24 Jul, 2017 and from 04 Nov, 2017 to 04 Oct, 2018. Sampling frequency of the signal is 32 kHz and it will be downsampled to 16 kHz in our dataset. Each recording is a 5 minutes audio file in WAV format. Each recording will be sliced into 100 segments to make up the input of neural networks. Namely, each segment has 3 seconds of duration.

These data in Ocean Networks Canada are acquired for disaster mitigation, ocean management and environment protection. It means that no target type labels are generated at the same time. Fortunately, the related references remind us that Automatic Identification System (AIS) nearby the hydrophone can record the information about passing targets approximately. Therefore, we choose the AIS information as the standard truth of targets to label audio samples. Concretely, we use the log recorded by Digital Yacht AISnet Receiver 1302-0097-01 (12522) deployed at Latitude $49.2160633333^{\circ }$, Longitude $-123.2054816667^{\circ }$ which is the closest receiver device to the hydrophone.

By querying the AIS record of corresponding time and area of a signal recording, we can acquire the passing targets information approximately. It is possible to use these data materials to manufacture an underwater target recognition dataset.

### Dataset manufacture

Firstly, targets presented in an area of 2 km radius of the hydrophone deployment site are selected. To minimize noise generated by other ships, the recording will be removed if there are other ships presented in 3 km radius of the hydrophone deployment site.

Then, according to the AIS information of the target, the Maritime Mobile Service Identify (MMSI) of the target is available. By querying the MMSI, the type of target can be confirmed, and corresponding recording can be annotated. In order to make the labeled categories as relevant as possible to the source of acoustic radiation, we adopt the label system in^8^. Combining with the collected data, the label system used in this paper is as follows:

- Class A: fishing boats, trawlers, mussel boats, tugboats and dredgers
- Class B: passenger ferries
- Class C: ocean liners and ro-ro vessels
- Class D: background noise recordings

Finally, dataset with 4 target categories is manufactured. However, the original dataset is imbalanced among categories and it is not conducive to training model. We randomly sample from the categories with large quantities of recordings. After sampling, the number of recordings is close between categories and is similar among each month. The number of recordings of each categories is shown in Table 1. Class A, C and D are obtained by random sampling. Each category has recordings about 62.5 hours. In order to get close to the real application situation, the recordings from the first 12 months are used for training and the remaining 5 months for testing. Segments from one recording can not be split into the training dataset and testing dataset concurrently. Table 1 also shows the partitioning details of dataset.

**Table 1:** The number of recordings.

Class label	A	B	C	D
Total number of recordings	750	750	750	750
Number of training recordings	530	538	530	530
Number of testing recordings	220	212	220	220

Each recording is a 5 min audio file in WAV format. Each recording will be sliced into 100 segments as samples to make up the input of neural networks. Each segment has 3 s duration.

## Results

The experiments are designed around two aspects. In the first experiment, the networks such as ResNet^45^, DRSN^47^ and DNN^48^ which have related structures and same input form with MSRDN will be used for comparison. By adding and modifying network structures step by step, the performance gains from the improvements are gradually reflected. Besides, in order to demonstrate that MSRDN can break through the limitation of the spectrogram-based methods, the networks such as ResNet^45^, Inception-Resnet^39^, DenseNet^46^ and the modified LENET (MLENET)^43^ which take the time-frequency representation as input will be used to compare with MSRDN.

In the second experiment, based on BigGAN^42^ and WaveGAN^41^, we modify the structures of two models by replacing the core convolutional layers by our MSRU and compare the performance. The second experiment is conducted because we want to demonstrate that the structure we proposed can actually capture the features of underwater targets, rather than just promote the classification indicators through training tricks. Therefore, we add the MSRU into the generative task for investigation. The generated data in second experiment will not be used in the previous experiments in this paper.

Evaluation indicators in this paper follows the general classification task and generative task. For classification task, accuracy, average recall, average precision, macro F1 score and AUC (Area Under Curve) are used to evaluate models. There are four categories in dataset to classify. The confusion matrix is shown in Table 2.

**Table 2:** Formal description of the confusion matrix.

Truth	Prediction			
	Class A	Class B	Class C	Class D
Class A	$A_{A}$	${A_{B}}$	$A_{C}$	$A_{D}$
Class B	$B_{A}$	$B_{B}$	$B_{C}$	$B_{D}$
Class C	$C_{A}$	$C_{B}$	$C_{C}$	$C_{D}$
Class D	$D_{A}$	$D_{B}$	$D_{C}$	$D_{D}$

Accuracy is defined as the ratio of identifying correct:

2 $$\begin{aligned} Accuracy = \frac{A_{A}+B_{B}+C_{C}+D_{D}}{n}, \end{aligned}$$

where *n* is the number of samples of test dataset.

For each class $k (k=A, B, C, D)$, recall, precision and F1 score are defined as:

3 $$\begin{aligned} recall_{k}= & {} \frac{k_{k}}{k_{A}+k_{B}+k_{C}+k_{D}}, \end{aligned}$$

4 $$\begin{aligned} precision_{k}= & {} \frac{k_{k}}{A_{k}+B_{k}+C_{k}+D_{k}}, \end{aligned}$$

5 $$\begin{aligned} F1\ score_{k}= & {} 2 \cdot \frac{precision_{k} \cdot recall_{k} }{precision_{k} + recall_{k}}, \end{aligned}$$

average recall, average precision and macro F1 score are calculated by average them among all categories correspondingly. AUC is the area under the Receiver Operating Characteristic (ROC) curves, and is often used to evaluate multiclassification models^18,19,28,37^. The closer the AUC is to 1, the better the model performance is.

For generative task, we mainly evaluate the performance of generator. Fréchet Inception Distance (FID)^51^ are used to measure the quality of generated samples. This indicator need to uses a pre-trained classifier. In our paper, the trained MSRDN model is used as the pre-trained classifier during the calculation process of FID. Based on deep feature maps of pre-trained classifier, FID calculates the distance between the real samples and the generated samples at the feature level. To be specific, FID assumes that if two samples are similar, their deep feature maps from the same classification model should also be similar. At the dataset level, if generated dataset is similar to testing dataset, the FID value between them should be low. In fact, two datasets can not be exactly the same. For the generator, it is meaningless to generate samples that are identical to the real dataset. In other words, we expect that the generated dataset can be similar to the real dataset and maintain some degree of diversity at the same time. For measuring this expectation accurately, we firstly calculate the FID between the training set and the testing set ($FID_{base}$). $FID_{base}$ represents the degree of diversity we expect, and the inherent differences in the real dataset itself. Ideally, FID between the real data and the generated data should be close to $FID_{base}$.

Then, we calculate the FID between the training set and the generated set ($FID_{trg}$) and the FID between the testing set and the generated set ($FID_{teg}$). Finally, we use *FID*(*TRG*) to measure the distance between the training set and the generated set, where *FID*(*TRG*) is the ratio value of $FID_{trg}$ and $FID_{base}$. Correspondingly, we use *FID*(*TEG*) to measure the distance between the testing set and the generated set, where *FID*(*TEG*) is the ratio value of $FID_{teg}$ and $FID_{base}$.

All experiments are conducted on a regular rack server with a Nvidia Titan RTX GPU (24G). Training and testing models are both based on the GPU. All neural networks are implemented on the open source machine learning framework pytorch-1.6.0^52^ under Linux operating system with python programming language.

### Classification experiments

A set of ablation experiments are designed to compare the performance of MSRDN and reference models, including ResNet^45^, DRSN^47^, DNN^48^. Besides, for the completeness of the ablation experiment, we design an intermediate model called Large kernel size with Soft-thresholding Deep Network (LSDN) which takes advantages of DRSN^47^ and DNN^48^ by combining large convolution kernel and soft threshold.

ResNet^45^ is a milestone model in deep learning. By adding shortcuts, the network can easily enjoy accuracy gains from greatly increased depth. We adopt the pre-activate version in this paper. The stacking configuration (the setting of layers number and channels number) of ResNet follows ResNet-101^45^, to make sure the depth of models and the resource consumption are in the similar level. In this experiment, ResNet will be modified to 1D version, by changing the function of convolution and pooling.

Based on ResNet, DRSN^47^ took soft thresholding which can learn the threshold automatically as nonlinear transformation layers into the deep architectures to eliminate unimportant features. Also, to make sure the depth of models and the resource consumption are in the similar level, we construct DRSN followed by the stacking configuration of ResNet-101^45^.

The third comparison model is deep neural network (DNN) used in^48^. Because the network in^48^ was not designed for underwater target recognition, we extend this network to follow the stacking configuration of ResNet-101^45^. The kernel sizes will be changed from 16 to 17 for convenient calculation. Compared with ResNet, DNN^48^ only increase the kernel size of convolution to change the receptive field of the model.

The fourth comparison model LSDN combines large convolution kernel and soft threshold. Compared with DNN^48^, Soft-thresholding is inserted as nonlinear transformation layers into the deep architectures in LSDN. Compared with DRSN^47^, the kernel size of the middle convolution layer is increased to 17 in every residual unit in LSDN.

In comparisons with T-F representation based models, the deep neural networks with well-designed stack structures such as ResNet^44,45^, Inception-Resnet^39^, DenseNet^46^ and the modified LENET (MLENET)^43^ are used to compare with our MSRDN.

The original 2D version ResNet^45^ will be adopted. Inception Series networks proposed by Google. The core idea is to achieve better perception by widening the networks. In each Inception module, convolution kernels of different sizes are used, which can be understood as different sensory fields, and then concentrate to enrich each layer of information. Inception has been shown to achieve good performance in image recognition at a relatively low computational cost. Inception-Resnet combined the structure of InceptionV4 and ResNet. DenseNet^46^ connects each layer to every other layer in a feed-forward fashion. This model alleviates the vanishing-gradient problem, strengthen feature propagation, encourage feature reuse, and substantially reduce the number of parameters. Besides, the MLENET^43^ achieved good results in underwater acoustic target recognition.

For ResNet of 2D version, we adopt the pre-activate version. The stacking configuration (the setting of layers number and channels number) of ResNet follows ResNet-101^45^. The parameters in Inception-Resnet that determine depth of network are the number of three modules. These are L, K and M. In this paper, they are taken as L = 5, K = 10, M = 5. For DenseNet^46^, we adopt DenseNet-201 model in our experiments.

**Figure 4:**
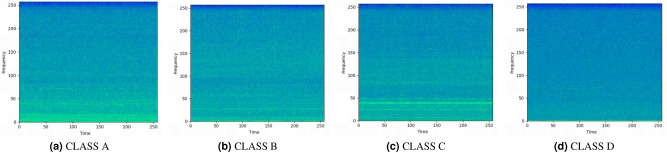
Time-Frequency Representation of each category.

For the generation of time-frequency spectrograms, a short time fast Fourier-transform with the frame length of 512 points (32ms) was used to yield the representation of the signal data. The hop length between frames is 187 points. Thus, the signal data was transformed into spectrogram of shape 257 × 257. Figure 4 shows the time-frequency representation generated by each category sample.

We train the networks from scratch, and use Swish^53^ as the activation function. We use the Adam optimizer^54^ with the default parameters. The Batch size is set to 64 for all experiments. The learning rate is from 1e−3 to 1e−8, and reduce as the training epoch increasing. We save the best model and evaluate on the testing set during the optimization process. The experimental results of classification experiments are shown in Table 3. The entire training process lasted 3–4 days to run through the whole training set about 80 times (80 epoch). Each model has been fully trained.

**Table 3:** Classification experimental results.

Input	Models	Accuracy (%)	Avg precision (%)	Avg recall (%)	Macro F1 (%)	AUC	Parameter size
Wave	ResNet^45^	76.16	76.18	76.06	76.08	0.9147	35.95M
Wave	DRSN^47^	80.30	80.20	80.20	80.15	0.9143	111.97M
Wave	DNN^48^	81.19	80.76	81.05	80.82	0.9278	61.61M
Wave	LSDN	81.99	82.02	81.91	81.94	**0**.**9408**	137.63M
Wave	MSRDN	**83**.**15**	**82**.**85**	**83**.**04**	**82**.**91**	0.9307	54.38M
T-F	Inception-Res^39^	82.38	82.26	82.32	82.17	0.9165	29.82M
T-F	DenseNet^46^	81.86	81.66	81.78	81.64	0.9293	18.09M
T-F	ResNet^45^	80.10	80.13	80.04	79.96	0.9111	42.49M
T-F	MLENET^43^	78.67	78.46	78.59	78.45	0.9297	3.19M

From experimental results of wave-based models, it can be seen that both Soft-thresholding and Large kernel size can significantly improve the performance 4–5% in accuracy compared with ResNet^45^ which can be considered as a basic deep convolutional stack. Compared with Soft-thresholding, Large kernel size can bring more performance improvement. Indeed, as the receptive field gets larger, the model learns more features. Whereas Soft-thresholding can only maximize the use of features based on the original receptive field. LSDN combining large convolution kernel and Soft-thresholding improve the performance 0.8%-1.69% in accuracy compared with DRSN^47^ and DNN^48^. It shows that the two methods improve the performance of the model from different perspective, and they can be used in combination without conflict. Compared with other models, MSRDN achieves the highest performance in accuracy, average precision, average recall and macro F1 score. To our surprise, LSDN achieves the highest AUC, and MSRDN achieves the second highest AUC. One possible reason is that AUC is affected by output distribution of softmax layer. Although MSRDN achieves better on accuracy, the output distribution of softmax layer may not be as sharp as LSDN. There is room for improvement in the confidence of MSRDN model. In terms of the parameters size, benefiting from to multiscale convolution, our model is only larger than ResNet of 1D version and smaller than other wave-based models.

From experimental results of spectrogram-based models, we can find out that our model achieves the highest recognition indicators compared with deep models which takes time-frequency representation as input. Compared with ResNet^45^, Inception-Resnet^39^ and DenseNet^46^, MSRDN improves 3.05%, 1.29% and 0.77% in accuracy respectively. Compared with MLENET^43^, the performance advantages of MSRDN are also obvious. However, because the convolutional kernels are small, the parameters sizes of spectrogram-based models are generally small. It is the advantage of spectrogram-based models. In addition, we observed that Inception-Resnet achieves higher accuracy and F1 score than ResNet of 2D version^45^ and DenseNet^46^. The reason may have to do with the shape of the spectrogram features. In T-F spectrogram, target features are usually in banded shape.

Specific to each category, the confusion matrix of the proposed model on test data is shown in Table 4. The accuracy is listed at the bottom-right corner. Both the precision and recall of Class C are higher than that of other classes. This is most likely due to the large differences between ocean liners and other categories. The high performance of background noise (Class D) demonstrates that it is easy to identify if there is no target. The confusion between class A and class B are larger than other categories. This reason may be that two categories occasionally have similar propulsion systems, gross tonnage and size.

**Table 4:** Confusion matrix of samples.

Truth	Prediction				
	Class A	Class B	Class C	Class D	Recall (%)
Class A	16944	3040	1368	648	77.02
Class B	3393	15027	574	2206	70.88
Class C	571	737	20554	138	93.43
Class D	352	1509	154	19985	90.84
Precision	79.70%	73.98%	90.75%	86.98%	83.15

The accuracy is listed at the bottom-right corner.

### Generative experiments

In generative experiments, we choose two advanced models BigGAN^42^ and WaveGAN^41^. Both of them are improvements of Deep Convolutional Generative Adversarial Network (DCGAN)^55^. We modify the structures of two models by replacing the core convolutional layers by our MSRU and compare the performance.

WaveGAN^41^ is a first application of GANs on unsupervised audio generation. Unlike the BigGAN, the original WaveGAN was an unsupervised model. It means that the label information is not being used. To align the models and make them comparable, we modify the two models to ACGAN^56^ form. In generator, labels will be combined with the input random vector after passing through an embedded layer. The label information is used to supervise the generator to generate samples for specified category. In discriminator, an auxiliary classifier is added to the tail of the network. The discriminator can not only distinguish the true and false samples, but also predict the sample category. The concrete structure of WaveGAN used in this paper can be found as Supplementary Tables S1 and S2 online.

For adding the MSRU into WaveGAN, we replace each convolution layer with MSRU structure and construct the MSRWaveGAN. For this purpose, we designed two basic units based on MSRU. These two types of units are used in generator and discriminator respectively. The concrete structure of basic unit used in generator and discriminator can be found as Supplementary Figs. S1 and S2 online. Finally, The concrete structure of MSRWaveGAN used in this paper can be found as Supplementary Tables S3 and S4 online.

BigGAN^42^ is an advanced generative adversarial network proposed by Google DeepMind. It uses the ResNet architecture for both generator and discriminator. In this experiment, BigGAN will be modified to 1D version, by changing the function of convolution, pooling, and upsampling layer. The structure of residual blocks used in generator and discriminator can be found as Supplementary Figs. S3 and S4 online. Overall, the concrete structure of BigGAN used in this paper can be found as Supplementary Tables S5 and S6 online.

Correspondingly, we create the MSBigGAN by using our MSRU to replace the residual block in BigGAN. The structure of residual blocks used in generator and discriminator can be found as Supplementary Figs. S5 and S6 online.

In terms of loss function, for the true-false discrimination, we use hinge loss just like BigGAN. For the auxiliary classifier, we use cross-entropy loss in common with general classification tasks. The Batch size is set to 64 for all experiments. We use Adam optimizer^54^ with $\beta _{1} = 0$ and $\beta _{2} = 0.999$ with a constant learning rate. For generator, we use $2 \times 10^{-4}$ as learning rate, and for discriminator, we use $5 \times 10^{-5}$ as learning rate. Spectral Normalization^57^ is used in both generator and discriminator. We train the networks from scratch. For model validation, we use the generator to generate 250 batches of samples for each category. Generated set contains 64000 samples in total for batch size 64. The entire training process lasted 5–6 days to run through the whole training set about 40 times (40 epoch). The experimental results of GAN experiments are shown in Table 5.

**Table 5:** Generative experimental results.

Model	FID(TEG)	FID(TRG)
WaveGAN	1.9131	2.3598
MSRWaveGAN	1.0589	2.1155
BIGGAN	9.7091	9.4453
MSBIGGAN	1.6397	2.6340

From experimental results, we can find out that adding MSRU to WaveGAN and BigGAN can improve performance. Specifically, for BigGAN, there are significant improvements in both FID(TEG) and FID(TRG). One possible reason is that the original BigGAN take the kernel size of 3. As we analyzed earlier in Methods section, the kernel size of 3 may be not the good choice for convolution on one-dimensional signal waveform. In comparison, original WaveGAN take the kernel size of 25 as the filter length. Hence, MSRU brings the BigGAN greater promotion than WaveGAN.

**Figure 5:**
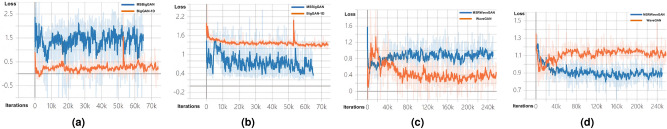
The loss curves of the discriminators and generators. (**a**) Generator loss of BigGAN and MSBigGAN. (**b**) Discriminator loss of BigGAN and MSBigGAN. (**c**) Generator loss of WaveGAN and MSRWaveGAN. (**d**) Discriminator loss of WaveGAN and MSRWaveGAN. The smoothing function is used, and the smoothing factors are set to 0.8 in all subfigure.

Besides, during training process, we observed an interesting phenomenon. When MSRUs are added in the two models, the loss of discriminator decreased obviously. Figure 5 shows the loss curves of the discriminators and generators. Because the generator and discriminator are in a dynamic game, the training process of GAN is usually unstable. Losses of networks are oscillating. Therefore, we use the smoothing function provided by TensorBoard^58^. We speculate that MSRUs enhance the discriminator more than the generator. The essential reason may be that signal synthesis and decomposition are based on different principles. The synthesis of signal is more like an additive process, whereas the decomposition of signal is more like an multiplication (filter) process. Therefore, multiscale feature extraction is more suitable for discriminator than generator. In fact, we believe that the improved discriminator can improve the performance of generator to some extent. Because the generator needs to be more powerful against the discriminator. On the other hand, this phenomenon also demonstrate the effectiveness of MSRUs in recognition.

## Discussion

Factors that will impact the recognition results are analyzed based on the process of making the dataset. Base error of dataset, label precision and time range need to be considered when modeling on the dataset. In fact, in underwater image and wireless signal quality assessment, quite a few methods had been published^59–62^. However, there are few evaluation methods of waveform data quality for underwater target recognition. Although it is difficult to give a quantitative method in quality assessment, we think it is necessary to analyze some influencing factors qualitatively.

### Base error of dataset

In this subsection, base error of the dataset are analyzed. Finding out the sources of error will facilitate the application of the dataset.

#### Target report missing-TRM

An obvious fact is that we cannot guarantee that every target will open the AIS transmitting device when passing near the hydrophone. The impact of such errors is wider. One case is that a recording labeled as background noise contains the ship-radiated noise of the targets. Another case is that a recording labeled as a category contains the ship-radiated noise of another type targets. Both cases could cause confusion in the dataset.

#### AIS parsing error-APE

Instead of clear text data, AIS recording data is stored in a special message format. Errors occur occasionally when the device receives information over wireless communications. Some AIS data parsing may be problematic. However, the impact of such errors is limited due to the send frequency of the messages. The sending frequency of AIS messages usually between a few and a dozen seconds, depending on the state of the ship. The probability of consecutive record error AIS messages over a long period is small.

#### Distance calculation error-DCE

In the process of making the dataset, the distance between target and hydrophone needs to be calculated by the target position reported in the AIS messages. As we know, the earth is not a regular sphere. There must be deviation in calculating the distance between two points based on the latitude and longitude. In fact, the distance we concerned is short. Although distance calculation error is inevitable, it is in a limited range.

#### Target annotation error-TAE

In the process of making the dataset, we found the target via the MMSI and annotate target according to its category. We could only rely on the query results of public website, to find out whether a MMSI corresponds to a specific ship. There may be several inaccurate information or mismatches. We used several different websites for the query, and this kind of error is minimized by cross check.

From above analysis, all base errors described may affect the recognition model. Errors from the dataset itself can be summarized as a base error level. This level of error reflects the quality of dataset. The higher the error level is, the worse the quality of the dataset is. Specifically, the Target Report Missing is the most influential error source. The only way to reduce this error is manual filtering. Recordings will be judged by expert experience.

### Label precision

In underwater acoustic target recognition, label precision is a key factor to impact recognition. Generally, the underwater acoustic target recognition is a weak label question. It means that there is no way to know exactly where and when the sound of a target starts and ends. Therefore, any annotation is approximate and must have a precision scale. In our dataset, the label precision is 5min. In other words, if there is a target in the five-minute recording, we annotated the recording as the target type. Naturally higher label precision makes the model recognition performance better but higher label precision also needs more costs on annotation.

### Time range

Time range represents the sample richness of the dataset. Marine environment changes periodically and the temperature and salinity of sea water will affect sound transmission. The longer the time range of the dataset, the more likely it is to include such variations, and the broader the applicability of the model. Therefore, the dataset in this paper chooses continuous 12 months of record for training, to include as much sample space as possible.

## Conclusion

In order to explore appropriate structure of deep convolution stacks for perceiving underwater acoustic radiated noise and give full play to the automatic feature learning and extraction capabilities of deep neural networks, a multiscale residual unit (MSRU) is proposed in this paper. Multiscale convolution is used to replace the original core convolution in ResNet with significant improvement of model receptive field. Based on MSRU, we present a multiscale residual deep neural network (MSRDN) to classify underwater acoustic target. MSRDN achieves the best recognition accuracy of 83.15%, improved by 6.99% from the structure related networks which take the original signal waveform as input and 4.48% from the networks which take the time-frequency representation as input. Classification and generative experiments have proved the effectiveness of the MSRU and MSRDN. The multiscale features can be perceived from the original signal waveform to improve the performance in underwater acoustic target recognition. Detailed analyses are correspondingly carried out to put forward several factors that will impact the recognition results.

In the future study, we will consider using the method of computer assistance or expert experience to reduce the base error. Then, pay more attention on analyze the trajectory information of each target in detail to improve the label precision. More information (like target shape, tonnage, power, etc.) will be used to optimize the annotation system and improve label correlation. Of course, more and more comprehensive real data needs to be collected.

In terms of model improvement, it is meaningful to consider how to fuse spectrum and waveform feature by deep neural network. If these two kinds of features can be complementary fused, the performance of the model will be greatly improved further. Besides, using the generator in GANs to extend the training dataset may be a an effective way to improve the generalization and robustness of recognition model. We will explore the influence of using different mixing apportion of real and generated data in training process. Besides, we are considering using NAS techniques to find the best parametric strategy for our model.

## Supplementary Information

Supplementary Information.
