# Supplementary material for: Deep convolution stack for waveform in underwater acoustic target recognition
Source: Sci Rep. 2021 May 5;11:9614. doi: 10.1038/s41598-021-88799-z (PMC8099869; doi:10.1038/s41598-021-88799-z)
Supplement: Supplementary file 1 — Supplementary Information. [file 41598_2021_88799_MOESM1_ESM.pdf]

Supplementary material

S1. Structures of WaveGAN and MSRWaveGAN

The structure of generator in WaveGAN is shown in Supplementary Table. S1. Generally, the Transposed Conv1D layer can be implemented in two ways. One uses the transpose convolution function directly. Another uses a one-dimensional convolution layer following an upsampling function. In this paper, we use the second implementation strategy, which is used by many models recently. The advantage of the second implementation is that the dimensions of output can be controlled precisely by setting the scale of upsampling or the size of upsampling.

Supplementary Table S1. The structure of generator in WaveGAN. *B* represents the batch size. The negative slope are 0.2 for all LeakyRelu layers

Operation	Operation Parameters	Input Shape	Output Shape
Input	Z $\sim Normal(0,1)$	——	[*B*,1,128]
Input	T $\sim RandomInt(0,3)$	——	[*B*,1]
Label Embedding	dimension=128	[*B*,1]	[*B*,1,128]
Tensor Concat	dim=2	[*B*,1,128]*2	[*B*,1,256]
Linear	in=256, out=2209	[*B*,1,256]	[*B*,1,2209]
Reshape	——	[*B*,1,2209]	[*B*,47,47]
LeakyRelu	negative_slope=0.2	[*B*,47,47]	[*B*,47,47]
Transposed Conv1D+LeakyRelu	out_c=32, kernel size=25, upsample_scale=4	[*B*,47,47]	[*B*,32,188]
Transposed Conv1D+LeakyRelu	out_c=16, kernel size=25, upsample_scale=4	[*B*,32,188]	[*B*,16,752]
Transposed Conv1D+LeakyRelu	out_c=8, kernel size=25, upsample_scale=4	[*B*,16,752]	[*B*,8,3008]
Transposed Conv1D+LeakyRelu	out_c=4, kernel size=25, upsample_scale=4	[*B*,8,3008]	[*B*,4,12032]
Transposed Conv1D+LeakyRelu	out_c=1, kernel size=25, upsample_size=48000	[*B*,4,12032]	[*B*,1,48000]
Tanh	——	[*B*,1,48000]	[*B*,1,48000]

The structure of discriminator in WaveGAN is shown in Supplementary Table. S2. Phase Shuffle layer is propsed in WaveGAN, and we do not change about it. In the end of network, classify linear layer with SoftMax is used to predict the sample categories and discriminate linear layer with Sigmoid is used to distinguish authenticity of samples.

Supplementary Table S2. The structure of discriminator in WaveGAN. *B* represents the batch size. The negative slope are 0.2 for all LeakyRelu layers

Operation	Operation Parameters	Input Shape	Output Shape
Input	——	——	[*B*,1,48000]
Conv1D+LeakyRelu	out_c=4,kernel size=25, stride=4	[*B*,1,48000]	[*B*,4,12000]
Phase Shuffle	factor=2	[*B*,4,12000]	[*B*,4,12000]
Conv1D+LeakyRelu	out_c=8,kernel size=25, stride=4	[*B*,4,12000]	[*B*,8,3000]
Phase Shuffle	factor=2	[*B*,8,3000]	[*B*,8,3000]
Conv1D+LeakyRelu	out_c=16,kernel size=25, stride=4	[*B*,8,3000]	[*B*,16,750]
Phase Shuffle	factor=2	[*B*,16,750]	[*B*,16,750]
Conv1D+LeakyRelu	out_c=32,kernel size=25, stride=4	[*B*,16,750]	[*B*,32,187]
Phase Shuffle	factor=2	[*B*,32,187]	[*B*,32,187]
Conv1D+LeakyRelu	out_c=47,kernel size=25, stride=4	[*B*,32,187]	[*B*,47,47]
Reshape	——	[*B*,47,47]	[*B*,2209]
Linear(classify)	in=2209,out=4	[*B*,2209]	[*B*,4]
SoftMax	——	[*B*,4]	[*B*,4]
Linear(discriminate)	in=2209,out=1	[*B*,2209]	[*B*,1]
Sigmoid	——	[*B*,1]	[*B*,1]

The structure of basic unit (MSRU_GBlock) used in generator of MSRWaveGAN is shown in Supplementary Fig. S1. Specifically, we remove the soft-activation layer, Considering that the generator is more about synthesizing signals than filtering them. The whole structure is still represented in the form of residual blocks. The structure of basic unit (MSRU_DBlock) used in discriminator of MSRWaveGAN is shown in Supplementary Fig. S2. Different from MSRU_GBlock, the upsampling

functions are replaced by global average pooling, and all batch normalization layers on the main and shortcut information paths are removed. These differentiated designs are used by many models recently.

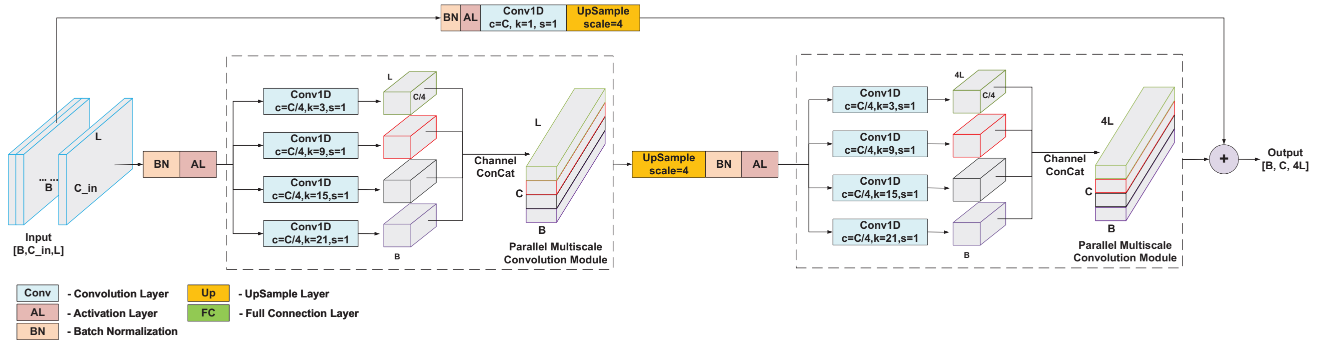

Supplementary Figure S1. The structure of basic unit used in generator (MSRU_GBlock) of MSRWaveGAN. The shape of input data is $[B, C\_in, L]$ in which B represents batch size, C_in represents channel number, and L represents data length. The shape of output data is $[B, C, L*4]$.

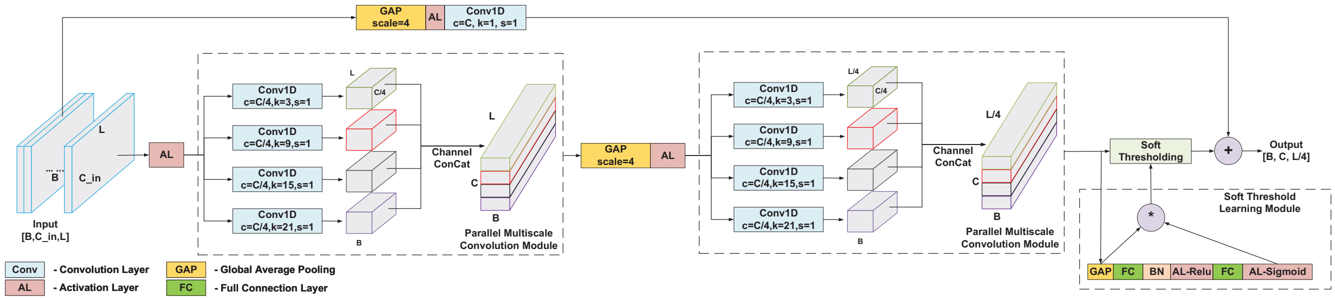

Supplementary Figure S2. The structure of basic unit used in discriminator (MSRU_DBlock) of MSRWaveGAN. The shape of input data is $[B, C\_in, L]$ in which B represents batch size, C_in represents channel number, and L represents data length. The shape of output data is $[B, C, L/4]$.

The structure of MSRWaveGAN is shown in Supplementary Table. S3 and Supplementary Table. S4. We replace each convolution layer with MSRU structure and construct the MSRWaveGAN.

Supplementary Table S3. The structure of generator in MSRWaveGAN. B represents the batch size.

Operation	Operation Parameters	Input Shape	Output Shape
Input	$Z \sim Normal(0,1)$	——	[B,1,128]
Input	$T \sim RandomInt(0,3)$	——	[B,1]
Label Embedding	dimension=128	[B,1]	[B,1,128]
Tensor Concat	dim=2	[B,1,128]*2	[B,1,256]
Linear	in=256, out=2209	[B,1,256]	[B,1,2209]
Reshape	——	[B,1,2209]	[B,47,47]
MSRU_GBlock	C=32,upsample_scale=4	[B,47,47]	[B,32,188]
MSRU_GBlock	C=16,upsample_scale=4	[B,32,188]	[B,16,752]
MSRU_GBlock	C=8,upsample_scale=4	[B,16,752]	[B,8,3008]
MSRU_GBlock	C=4,upsample_scale=4	[B,8,3008]	[B,4,12032]
MSRU_GBlock	C=4,upsample_size=48000	[B,4,12032]	[B,4,48000]
BatchNorm+LeakyRelu	negative_slope=0.2	[B,4,48000]	[B,4,48000]
Conv1D	out_c=1,kernel size=1,stride=1	[B,4,48000]	[B,1,48000]
Tanh	——	[B,1,48000]	[B,1,48000]

Supplementary Table S4. The structure of discriminator in MSRWaveGAN. B represents the batch size.

Operation	Operation Parameters	Input Shape	Output Shape
Input	——	——	[B,1,48000]
MSRU_DBlock	C=4,GAP_scale=4	[B,1,48000]	[B,4,12000]
Phase Shuffle	factor=2	[B,4,12000]	[B,4,12000]
MSRU_DBlock	C=8,GAP_scale=4	[B,4,12000]	[B,8,3000]
Phase Shuffle	factor=2	[B,8,3000]	[B,8,3000]
MSRU_DBlock	C=16,GAP_scale=4	[B,8,3000]	[B,16,750]
Phase Shuffle	factor=2	[B,16,750]	[B,16,750]
MSRU_DBlock	C=32,GAP_scale=4	[B,16,750]	[B,32,187]
Phase Shuffle	factor=2	[B,32,187]	[B,32,187]
MSRU_DBlock	C=47,GAP_scale=4	[B,32,187]	[B,47,47]
LeakyRelu	negative_slope=0.2	[B,47,47]	[B,47,47]
Reshape	——	[B,47,47]	[B,2209]
Linear(classify)	in=2209,out=4	[B,2209]	[B,4]
SoftMax	——	[B,4]	[B,4]
Linear(discriminate)	in=2209,out=1	[B,2209]	[B,1]
Sigmoid	——	[B,1]	[B,1]

S2. Structures of BigGAN and MSBigGAN

The structure of residual block (GBlock) used in generator of BigGAN is shown in Supplementary Fig. S3. Class-conditional batch normalization layers are used just like BigGAN. The structure of residual block (DBlock) used in discriminator of BigGAN is shown in Supplementary Fig. S4. The implementations of two structures are consistent with BigGAN. The function of convolution, pooling, and upsampling layers are changed from 2D version to 1D version.

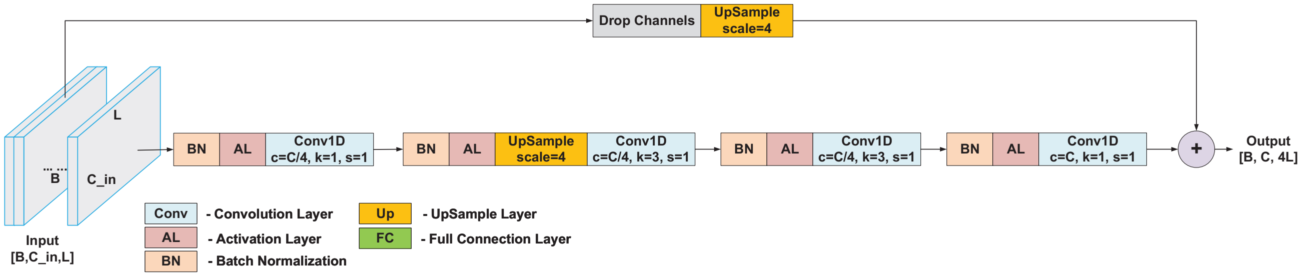

Supplementary Figure S3. The structure of residual block used in generator (GBlock) of BigGAN. The shape of input data is $[B, C\_in, L]$ in which B represents batch size, C_in represents channel number, and L represents data length. The shape of output data is $[B, C, L*4]$.

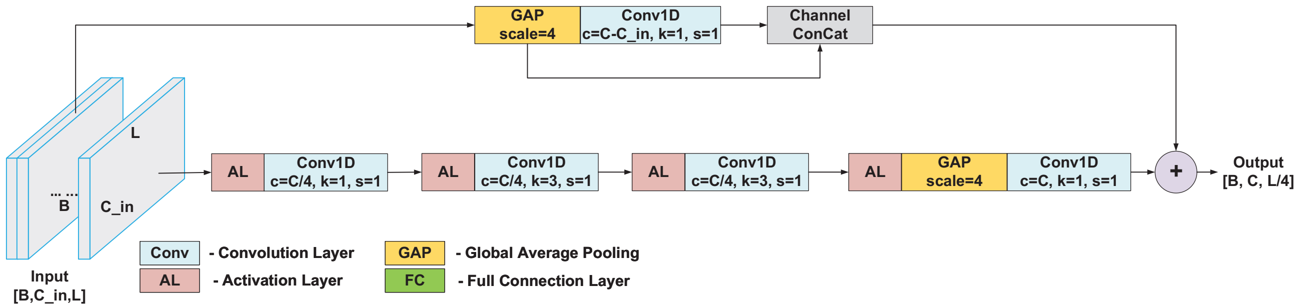

Supplementary Figure S4. The structure of residual block used in discriminator (DBlock) of BigGAN. The shape of input data is $[B, C\_in, L]$ in which B represents batch size, C_in represents channel number, and L represents data length. The shape of output data is $[B, C, L/4]$.

Overall, the concrete structure of BigGAN used in this paper is shown in Supplementary Table. S5 and Supplementary Table. S6.

The structure of residual blocks (MSGBlock) used in generator is shown in Supplementary Fig. S5. The structure of residual blocks (MSDBlock) used in discriminator is shown in Supplementary Fig. S6. Same as before, we remove the soft-activation layer in MSGBlock, Considering that the generator is more about synthesizing signals than filtering them. Replacing GBlock with MSGBlock and replacing DBlock with MSDBlock in BigGAN, we can construct MSBigGAN.

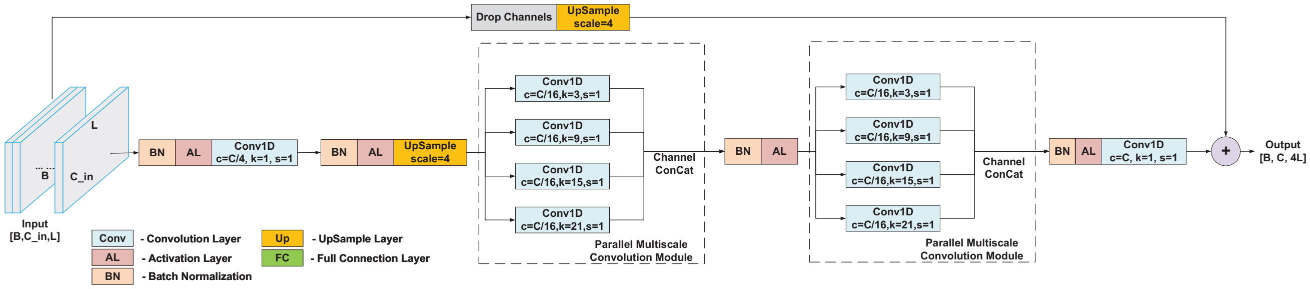

Supplementary Figure S5. The structure of residual block used in generator (MSGBlock) of MSBigGAN. The shape of input data is $[B, C\_in, L]$ in which B represents batch size, C_in represents channel number, and L represents data length. The shape of output data is $[B, C, L*4]$.

Supplementary Table S5. The structure of generator in BigGAN. B represents the batch size.

Operation	Operation Parameters	Input Shape	Output Shape
Input	$Z \sim Normal(0,1)$	——	[B,1,128]
Input	$T \sim RandomInt(0,3)$	——	[B,1]
Label Embedding	dimension=128	[B,1]	[B,1,128]
Tensor Concat	dim=2	[B,1,128]*2	[B,1,256]
Linear	in=256, out=2048	[B,1,256]	[B,1,2048]
Reshape	——	[B,1,2048]	[B,2048,1]
Upsample	upsample_scale=3	[B,2048,1]	[B,2048,3]
GBlock	out_c=2048,upsample_scale=False	[B,2048,3]	[B,2048,3]
GBlock	out_c=2048,upsample_scale=4	[B,2048,3]	[B,2048,12]
GBlock	out_c=2048,upsample_scale=False	[B,2048,12]	[B,2048,12]
GBlock	out_c=1024,upsample_scale=4	[B,2048,12]	[B,1024,48]
GBlock	out_c=1024,upsample_scale=False	[B,1024,48]	[B,1024,48]
GBlock	out_c=512,upsample_scale=4	[B,1024,48]	[B,512,192]
GBlock	out_c=512,upsample_scale=False	[B,512,192]	[B,512,192]
GBlock	out_c=256,upsample_scale=4	[B,512,192]	[B,256,768]
Non-Local Block	C=256	[B,256,768]	[B,256,768]
GBlock	out_c=256,upsample_scale=False	[B,256,768]	[B,256,768]
GBlock	out_c=128,upsample_scale=4	[B,256,768]	[B,128,3072]
GBlock	out_c=128,upsample_scale=False	[B,128,3072]	[B,128,3072]
GBlock	out_c=64,upsample_scale=4	[B,128,3072]	[B,64,12288]
GBlock	out_c=64,upsample_scale=False	[B,64,12288]	[B,64,12288]
GBlock	out_c=32,upsample_size=48000	[B,64,12288]	[B,32,48000]
BatchNorm+LeakyRelu	negative_slope=0.2	[B,32,48000]	[B,32,48000]
Conv1D	out_c=1,kernel size=3,stride=1	[B,32,48000]	[B,1,48000]
Tanh	——	[B,1,48000]	[B,1,48000]

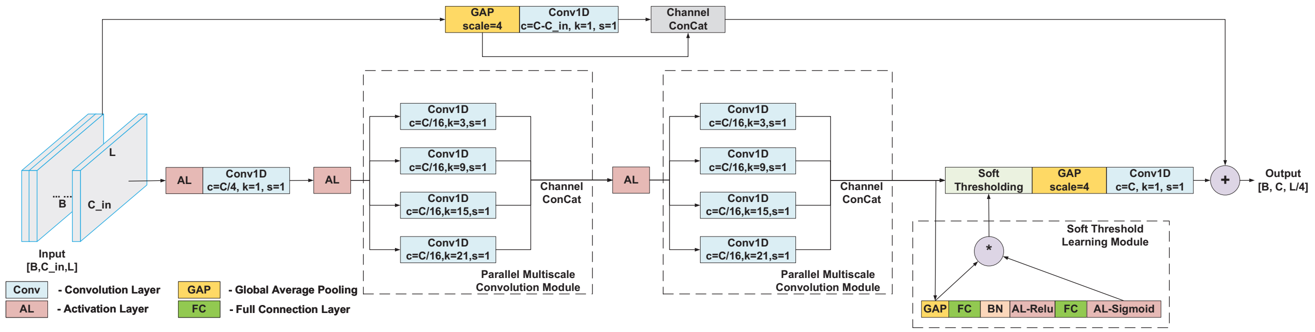

Supplementary Figure S6. The structure of residual block used in discriminator (DBlock) of MSBigGAN. The shape of input data is $[B, C\_in, L]$ in which B represents batch size, C_in represents channel number, and L represents data length. The shape of output data is $[B, C, L/4]$.

Supplementary Table S6. The structure of discriminator in BigGAN. *B* represents the batch size.

Operation	Operation Parameters	Input Shape	Output Shape
Input	——	——	[*B*,1,48000]
Conv1D	out_c=32,kernel size=3,stride=1	[*B*,1,48000]	[*B*,32,48000]
DBlock	out_c=64,GAP_size=12288	[*B*,32,48000]	[*B*,64,12288]
DBlock	out_c=64,GAP_scale=False	[*B*,64,12288]	[*B*,64,12288]
DBlock	out_c=128,GAP_scale=4	[*B*,64,12288]	[*B*,128,3072]
DBlock	out_c=128,GAP_scale=False	[*B*,128,3072]	[*B*,128,3072]
DBlock	out_c=256,GAP_scale=4	[*B*,128,3072]	[*B*,256,768]
DBlock	out_c=256,GAP_scale=False	[*B*,256,768]	[*B*,256,768]
Non-Local Block	C=256	[*B*,256,768]	[*B*,256,768]
DBlock	out_c=512,GAP_scale=4	[*B*,256,768]	[*B*,512,192]
DBlock	out_c=512,GAP_scale=False	[*B*,512,192]	[*B*,512,192]
DBlock	out_c=1024,GAP_scale=4	[*B*,512,192]	[*B*,1024,48]
DBlock	out_c=1024,GAP_scale=False	[*B*,1024,48]	[*B*,1024,48]
DBlock	out_c=2048,GAP_scale=4	[*B*,1024,48]	[*B*,2048,12]
DBlock	out_c=2048,GAP_scale=False	[*B*,2048,12]	[*B*,2048,12]
DBlock	out_c=2048,GAP_scale=4	[*B*,2048,12]	[*B*,2048,3]
DBlock	out_c=2048,GAP_scale=False	[*B*,2048,3]	[*B*,2048,3]
LeakyRelu	negative_slope=0.2	[*B*,2048,3]	[*B*,2048,3]
Global Sum Pooling	——	[*B*,2048,3]	[*B*,2048]
Linear(classify)	in=2048,out=4	[*B*,2048]	[*B*,4]
SoftMax	——	[*B*,4]	[*B*,4]
Linear(discriminate)	in=2048,out=1	[*B*,2048]	[*B*,1]
Sigmoid	——	[*B*,1]	[*B*,1]
